# Cross‐Cultural Adaptation and Psychometric Evaluation of the Turkish Version of the Exercise‐Specific Parkinson's Disease Questionnaire

**DOI:** 10.1002/brb3.71447

**Published:** 2026-06-10

**Authors:** Melis Yalçın, Turhan Kahraman, Berril Dönmez Çolakoğlu, Arzu Genç

**Affiliations:** ^1^ Graduate School of Health Sciences Dokuz Eylul University Izmir Türkiye; ^2^ Department of Physiotherapy and Rehabilitation, Faculty of Health Sciences Izmir Katip Celebi University Izmir Türkiye; ^3^ Department of Neurology Faculty of Medicine Dokuz Eylül University Izmir Türkiye; ^4^ Faculty of Physical Therapy and Rehabilitation Dokuz Eylül University Izmir Türkiye

**Keywords:** exercise, Parkinson, reliability, Turkish, validity

## Abstract

**Introduction:**

The Exercise‐Specific Parkinson's Disease Questionnaire (PDQ‐Exercise) is the first patient‐reported outcome measure to assess exercise‐related challenges in people with Parkinson's disease (pwPD). The aim was to translate and culturally adapt the PDQ‐Exercise into Turkish and to evaluate its psychometric properties in pwPD, including structural and construct validity assessed through predefined hypotheses regarding expected relationships with related measures, internal consistency, test–retest reliability, measurement error, and floor/ceiling effects.

**Methods:**

This cross‐cultural adaptation and psychometric validation study involved translating, culturally adapting, and evaluating the psychometric properties of PDQ‐Exercise into Turkish. Seventy pwPD were recruited. Structural validity was examined using confirmatory factor analysis (CFA). Construct validity was assessed through correlations with clinical, participation, and quality of life measures. Reliability was assessed through evaluations of internal consistency (Cronbach's alpha and McDonald's omega) and test–retest reliability (ICC) with standard error of measurement (SEM), minimal detectable difference (MDD), and Bland–Altman analysis.

**Results:**

The initial CFA model demonstrated poor fit to the data. Following theoretically and statistically justified modifications, including the addition of correlated residuals between selected items, model fit improved substantially. The final model showed good fit indices (CFI = 0.991, TLI = 0.981, SRMR = 0.051), although the RMSEA confidence interval indicated some uncertainty (RMSEA = 0.051, 90% CI: 0.00–0.145). Construct validity was supported by moderate correlations between PDQ‐Exercise and Movement Disorder Society Unified Parkinson's Disease Rating Scale (ρ = 0.545), Oxford Participation and Activities Questionnaire (ρ = 0.514), and Parkinson's Disease Questionnaire‐8 (ρ = 0.494). PDQ‐Exercise demonstrated acceptable internal consistency (Cronbach's alpha = 0.783 and McDonald's omega = 0.788) and excellent test–retest reliability (ICC = 0.985, SEM = 2.6; and MDD = 7.2). The Turkish version of the PDQ‐Exercise exhibited a significant floor effect. 28.6% of participants scored the minimum score (0) on the total scale.

**Conclusion:**

The Turkish version of the PDQ‐Exercise demonstrated acceptable psychometric properties in a sample predominantly consisting of individuals with early‐stage PD.

## 1 Introduction

Parkinson's disease (PD) is a common, progressive neurodegenerative disorder characterized by motor and non‐motor symptoms (Balestrino and Schapira 2020; Halli‐Tierney et al. 2020). While diagnosis primarily relies on motor features, non‐motor symptoms are prevalent and substantially contribute to disease burden. PD management focuses mainly on symptom control through dopaminergic treatments, yet significant functional impairments often persist despite medical and surgical advances (Balestrino and Schapira 2020). Consequently, physiotherapy and rehabilitation are essential components of care, with evidence showing that individual, group‐based, and community‐based exercise programs can meaningfully improve functional performance (Jagadeesan et al. 2017; Magrinelli et al. 2016; Rafferty et al. 2021).

Among the various physiotherapy and rehabilitation strategies employed in PD, exercise is considered the cornerstone for addressing both motor and non‐motor symptoms (de Almeida et al. 2025; Magrinelli et al. 2016; Peng et al. 2024; Song et al. 2025; Wang et al. 2025). Aerobic exercises targeting large muscle groups (e.g., treadmill training, Nordic walking), moderate‐to‐high intensity resistance training, breathing exercises, and mind–body approaches such as yoga, tai chi, qigong, and tango have been shown to contribute to disease management (de Almeida et al. 2025; Ellis et al. 2021; Liu et al. 2019; Magrinelli et al. 2016; Peng et al. 2024; Pereira et al. 2019; Song et al. 2025; Wang et al. 2025; Yang et al. 2022).

In recent years, patient‐reported outcome measures (PROMs) have gained importance in PD research by capturing patients’ perspectives on disease impact and health‐related quality of life (de la Cuadra‐Grande et al. 2025; Yetiş et al. 2025). To address the lack of a tool focusing specifically on exercise‐related challenges, the Exercise‐Specific Parkinson's Disease Questionnaire (PDQ‐Exercise) was developed in 2021 (Morley et al. 2021). This seven‐item PROM, originally in English, has shown good psychometric properties and sensitivity to health deterioration in people with PD (Morley et al. 2021; Morley et al. 2022), supporting its suitability for research and clinical trials. Conceptually, the PDQ‐Exercise is intended to capture patients’ perceptions of exercise‐related difficulties and the impact of PD on their ability to engage in physical activity. Rather than assessing physical performance directly, the instrument reflects the subjective experience of barriers and limitations to exercise participation among people with PD. Clarifying this latent construct is important for structural validity testing. Confirmatory factor analysis (CFA) is used to test whether the observed items adequately represent this underlying dimension. However, its unidimensional structure was initially explored using only exploratory factor analysis (EFA) (Morley et al. 2021). In addition, the initial validation study did not evaluate certain measurement properties, such as test–retest reliability and measurement error, highlighting the need for further validation studies in different populations and languages (Morley et al. 2021). Therefore, cross‐cultural adaptation and comprehensive psychometric evaluation, including test–retest reliability and CFA, are needed to ensure that the PDQ‐Exercise accurately measures the intended construct in different populations.

Although the PDQ‐Exercise has been translated into Australian English, Brazilian Portuguese, and German (Oxford University 2023), information regarding the translation and validation processes of these versions is limited. Formal psychometric validation studies in these language versions have not yet been reported. When a questionnaire is intended for use in a different language or cultural setting, a rigorous cross‐cultural adaptation and validation process is essential. This is because linguistic and cultural nuances may alter the interpretation of items, potentially affecting the reliability and validity of the measure (Efstathiou 2018). A formal validation study ensures that the translated version measures the same construct as the original and is both reliable and valid in the target population (Fenn et al. 2020). Use of unvalidated translated instruments may lead to inaccurate conclusions.

The aim of this study was to translate and culturally adapt the PDQ‐Exercise into Turkish and to evaluate its psychometric properties in people with PD, including structural and construct validity (based on predefined hypotheses regarding expected relationships with related measures), internal consistency, test–retest reliability, measurement error, and floor and ceiling effects.

## 2 Methods

### 2.1 Study Design and Ethical Considerations

This study was designed as a cultural adaptation and psychometric validation study. The study was conducted and reported following the COSMIN (COnsensus‐based Standards for the selection of health Measurement INstruments) reporting checklist for patient‐reported outcome measures (Mokkink et al. 2019). The study protocol was approved by the Non‐Invasive Research Ethics Committee of Dokuz Eylul University (protocol number: 7071‐GOA). Since this study did not involve any intervention, treatment allocation, or prospective manipulation of variables, it was not pre‐registered in an open‐access repository.

Data were collected through face‐to‐face interviews between May 2022 and November 2023. All participants provided written informed consent before participating in the study. Data were collected during patients’ routine outpatient clinic visits. To assess test–retest reliability, participants were asked to return 7–14 days after the initial assessment to complete the PDQ‐Exercise again. A 7–14 day‐interval is commonly used in psychometric studies to avoid recall bias while minimizing the likelihood of clinical change (Marx et al. 2003).

### 2.2 Participants

Participants were recruited from the Movement Disorders Outpatient Clinic at the Department of Neurology, Dokuz Eylul University Hospital, representing a broad range of age, disease duration, and motor severity among people with PD attending this tertiary care center. The clinic is located in the third‐largest city in Izmir, Türkiye and serves as a reference center, receiving patients not only from the local area but also from other cities across the region. Sample size for test–retest reliability was calculated assuming an expected ICC of 0.85, a precision of ±0.10, and a 95% confidence level (Bonett 2002). With two measurements per participant and a 15% anticipated dropout, a minimum of 31 participants was required, yielding a target of 37. For construct validity, an a priori power analysis in G*Power (v3.1) (Faul et al. 2007), based on *r* = 0.50, α = 0.05, and 80% power, indicated a required sample of 29 participants. However, according to COSMIN guidelines, a sample size of 50–99 participants is considered adequate for evaluating most psychometric properties. For factor analysis, the recommended sample size is at least five times the number of items with a minimum of 100 participants, or alternatively, at least six times the number of items when the total sample size is below 100 (Mokkink et al. 2019). Accordingly, given the seven‐item PDQ‐Exercise, a minimum of 42 participants was required. In addition, commonly used rules of thumb suggest 5–10 participants per item (i.e., 35–70 for the PDQ‐Exercise) (Bryman and Cramer 2004); however, these guidelines are generally considered less robust than COSMIN recommendations. Overall, the study aimed to recruit a total of 70 participants.

Inclusion criteria were: age ≥40 years, diagnosis of idiopathic PD, Revised Turkish version of Mini‐Mental State Examination (rMMSE‐T) score >22 for literate participants and >18 for illiterate participants, history of at least 1 month of exercise therapy. Patients experiencing an “off” phase during assessment were excluded (Keskinoglu et al. 2009). The attending neurologist determined motor fluctuation (on/off) status.

### 2.3 Translation and Cross‐Cultural Adaptation Process

The Turkish translation and cultural adaptation of the PDQ‐Exercise were conducted in accordance with guidelines provided by Outcomes at Oxford University Innovation, with a responsible person from this company overseeing the entire process. After initial forward translation by two independent bilingual translators (native Turkish speakers), discrepancies were discussed and resolved by an expert committee to ensure conceptual and semantic equivalence, and a consensus Turkish version was produced. Two additional independent translators, native English speakers fluent in Turkish, then performed back‐translations of the Turkish version. The expert committee reviewed the back‐translated versions for consistency with the original English questionnaire. The reconciled version was submitted to the original developers for approval. Feedback was incorporated to produce the final Turkish version for pilot testing. Content validity was qualitatively assessed through expert committee review, during which all items were evaluated for clarity, relevance, and cultural appropriateness. Through discussion and iterative revisions, consensus was reached for all items, with full agreement among committee members on the final version of the instrument.

Cognitive debriefing was conducted through face‐to‐face interviews to evaluate the clarity, comprehensibility, and cultural relevance of the translated questionnaire in a sample of 15 people with PD. Participants ranged in age from 52 to 84 years, with disease duration varying between 3 and 16 years. The sample included both male and female participants with diverse educational backgrounds (from primary school to university level), occupations, and marital statuses. Detailed demographic characteristics of the pilot sample are provided in the Supporting Information. This variation in demographic and clinical characteristics enabled the assessment of item comprehension, clarity, and cultural appropriateness across a heterogeneous group. Participants were first asked to complete the pilot version of the instrument, and the time required to complete it was recorded by a researcher. Following completion, a structured interview was conducted using cognitive probing techniques. Participants were asked to comment on the response options, identify any items that were difficult to understand, and suggest alternative wording where necessary. In addition, they were asked to explain each item in their own words to assess item interpretation and conceptual equivalence. Feedback obtained from these interviews was used to refine the final version of the questionnaire.

### 2.4 Collected Data Regarding Demographic and Clinical Characteristics

Demographic and clinical data were collected, including age, sex, education level, and use of mobility assistive devices. The following clinical assessments were carried out by the same neurologist, a professor specializing in movement disorders.

#### 2.4.1 Revised Turkish Version of Mini‐Mental State Examination (rMMSE‐T)

A widely used screening tool for cognitive impairment, comprising 11 items across five domains and scored out of 30 (Keskinoglu et al. 2009; Molloy et al. 1991). Participants with scores above 22 for literate and above 18 for illiterate individuals were included in this study (Keskinoglu et al. 2009).

#### 2.4.2 Modified Hoehn and Yahr Stage (MHYS)

It was used to assess disease severity based on motor function and functional disability. Stages range from 1 (unilateral involvement) to 5 (bedridden or wheelchair‐bound) (Goetz et al. 2004).

#### 2.4.3 Movement Disorders Society Unified Parkinson's Disease Rating Scale (MDS‐UPDRS)

It is a comprehensive tool that assesses mentation, behavior, mood, activities of daily living, motor examination, and treatment complications across 42 items, each scored from 0 to 4. Higher scores indicate greater disability (Movement Disorder Society Task Force on Rating Scales for Parkinson's Disease 2003). This study utilized the Turkish version of MDS‐UPRDS, which possesses satisfactory psychometric properties (Akbostanci et al. 2018).

### 2.5 Target Scale and Parallel Scales to Assess Validity

#### 2.5.1 PDQ‐Exercise

It is a 7‐item, 5‐point Likert‐type questionnaire designed to assess exercise‐related challenges in people with PD. Scores are calculated on a 0–100 scale, with higher scores indicating greater exercise‐related difficulties (Morley et al. 2021).

#### 2.5.2 Oxford Participation and Activities Questionnaire (Ox‐PAQ)

It is a 23‐item PROM comprising three domains—routine activities, social engagement, and emotional well‐being. Each item is scored on a 5‐point Likert scale, and total scores range from 0 to 100, with higher scores indicating greater impairment (Morley et al. 2016). This study utilized the Turkish Ox‐PAQ, a reliable and valid questionnaire for assessing participation and activity levels (Karapinar et al. 2020).

#### 2.5.3 Parkinson's Disease Questionnaire‐8 (PDQ‐8)

It is a short‐form version of the PDQ‐39 assessing health‐related quality of life in people with PD. Scores range from 0 to 100, with higher scores indicating lower health‐related quality of life (Peto et al. 1995). The Turkish version of PDQ‐8 was found to be reliable and valid (Kahraman et al. 2018).

All questionnaires were completed by the participants themselves. A researcher was present to provide clarification if needed, but did not influence or record participants’ responses.

### 2.6 Statistical Analysis

Analyses were performed using IBM SPSS Statistics (Version 25.0. Armonk, NY: IBM Corp.) and Jamovi (version 2.5.4.0). The normality of the data was assessed using both statistical and graphical methods. Given that the sample size exceeded 50 participants, the Kolmogorov–Smirnov test was used. Additionally, skewness and kurtosis statistics were reviewed. To complement these statistical tests, graphical evaluations were performed. Histograms and boxplots were examined. Descriptive statistics, including medians (Q1‐Q3) for continuous variables and frequencies (%) for categorical variables, were used to summarize the participants’ demographic and clinical characteristics. A *p*‐value <0.05 was considered statistically significant.

Confirmatory factor analysis (CFA) was conducted to evaluate the structural validity of the Turkish version of the PDQ‐Exercise. Model fit was assessed based on several widely accepted fit indices: the Chi‐square test (χ^2^), Comparative Fit Index (CFI), Tucker–Lewis Index (TLI), Standardized Root Mean Square Residual (SRMR), and Root Mean Square Error of Approximation (RMSEA) with 90% confidence intervals. Model fit was considered acceptable based on the following thresholds: CFI and TLI≥0.95, SRMR≤0.08, and RMSEA≤0.06, with the upper bound of the 90% CI not exceeding 0.10 (Hu and Bentler 1999). Modification indices were examined to identify potential sources of model misfit. Where theoretically justified, post hoc error covariances between residuals of related items were added to improve model fit.

Hypothesis testing for construct validity was evaluated by examining Spearman correlations between the PDQ‐Exercise and MHYS, UPDRS, Ox‐PAQ, and PDQ‐8, due to non‐normal data distribution. Spearman correlation coefficients were computed to examine the strength and direction of the associations. The absolute magnitude of Spearman's rho (ρ) was interpreted according to the following classification: 0.00–0.10, negligible correlation; 0.10–0.39, weak correlation; 0.40–0.69, moderate correlation; 0.70–0.89, strong correlation; and 0.90–1.00, very strong correlation (Akoglu 2018; Schober et al. 2018). It was hypothesized that the PDQ‐Exercise would demonstrate a moderate correlation with the MHYS, as disease severity is one of the key determinants of exercise limitations in PD. Similarly, it was hypothesized that the PDQ‐Exercise would show at least moderate positive correlations with the MDS‐UPDRS Total, Motor Examination, and Motor Daily Living subscales, as these domains are closely related to motor function and, consequently, exercise limitations. In contrast, weak positive correlations were expected with the MDS‐UPDRS Non‐Motor Symptoms and Motor Complications subscales, as motor complications primarily reflect treatment‐related phenomena rather than functional motor capacity. Furthermore, it was hypothesized that the PDQ‐Exercise would demonstrate moderate‐to‐strong correlations with all Ox‐PAQ subscales, given that the Ox‐PAQ directly assesses physical activity participation, which conceptually overlaps with the construct captured by the PDQ‐Exercise. A moderate‐to‐strong correlation was also expected between the PDQ‐Exercise and the PDQ‐8, as both instruments belong to the PDQ family and assess related aspects of patient‐reported quality of life while remaining conceptually distinct. Hypothesis testing for construct validity was evaluated by calculating the proportion of confirmed hypotheses among the 11 a priori‐defined hypotheses.

Internal consistency was evaluated using Cronbach's alpha and McDonald's omega for the PDQ‐Exercise total and subscales, with values >0.80 considered very good, 0.70–0.79 acceptable, and <0.70 inadequate (36). Test–retest reliability was assessed using ICCs with 95% confidence intervals, calculated with a two‐way mixed‐effects model and absolute agreement (Koo and Li 2016). ICCs were interpreted as very low (≤0.25), low (0.26–0.49), moderate (0.50–0.69), high (0.70–0.89), or very high (≥0.90) (Munro 2005). Absolute reliability was examined using the SEM and MDD, calculated as SEM = SD × √(1−ICC) and MDD = SEM×1.96 × √2 (Charter 1996; Weir 2005). Agreement between test and retest scores was further evaluated using Bland–Altman analysis, reporting mean bias and 95% limits of agreement. The presence of proportional bias was examined using linear regression analysis, with the difference scores (test–retest) as the dependent variable and the mean of the two measurements as the independent variable.

To evaluate potential floor and ceiling effects, the proportions of participants scoring at the lowest and highest possible scores on each item were determined. A value exceeding 15% at either end was considered indicative of a floor or ceiling effect (McHorney and Tarlov 1995).

## 3 Results

A total of 70 participants were included in the study. The mean age of the participants was 67.77 ± 10.62 years. Most participants were male (57.1%). Results regarding participants’ cognitive status, disease severity, activity and participation, and health‐related quality of life are presented in Table 1.

**TABLE 1:** Participant characteristics and assessment scores (*n* = 70).

Variable	Median (IQR) or *n* (%)
Age, years	68 (59.8–76.3)
Sex	
Male	40 (57.1)
Female	30 (42.9)
Education	
Primary school	31 (44.2)
Secondary school	4 (5.7)
High school	12 (17.1)
University‐level	23 (32.9)
Users of assistive devices for walking	13 (18.6)
Disease duration, years	6 (3–10)
rMMSE‐T	26 (24–28)
MHYS	
Stage 1	14 (20.0)
Stage 1.5	15 (21.4)
Stage 2	26 (37.1)
Stage 2.5	4 (5.7)
Stage 3	8 (11.4)
Stage 4	2 (2.9)
Stage 5	1 (1.4)
MDS‐UPDRS	
Total	42.50 (32–55.25)
Non‐motor Symptoms	3 (2–5)
Motor Aspects of Daily Living	13 (8.75–16.25)
Motor Examination	22 (14–33)
Motor Complications	4 (2–6)
PDQ‐Exercise (0–100)	14.28 (0–28.57)
Ox‐PAQ (0–100)	
Total	21.5 (8.75–40.25)
Routine Activities	14.5 (6–26.25)
Social Engagement	2 (0–5)
Emotional Wellbeing	5 (2–10.25)
PDQ‐8 (0–100)	18.75 (9.37–34.37)

Abbreviations: IQR = interquartile range; MDS‐UPDRS = Movement Disorder Society Unified Parkinson's Disease Rating Scale; MHYS = Modified Hoehn and Yahr Stage; Ox‐PAQ = Oxford Participation and Activities Questionnaire; PDQ‐8 = Parkinson's Disease Questionnaire‐8; rMMSE‐T = Revised Turkish version of Mini‐Mental State Examination, PDQ‐Exercise = Parkinson's Disease Questionnaire‐Exercise.

The initial CFA model demonstrated poor fit to the data, χ^2^(14) = 115, *p* < 0.001. Additional fit indices also indicated inadequate fit, with CFI = 0.498, TLI = 0.247, RMSEA = 0.321 (90% CI: 0.268–0.376), and SRMR = 0.145. Standardized factor loadings in the initial model were all statistically significant (*p* < 0.01), ranging from 0.275 to 0.972, although some items (e.g., Item 7) showed relatively low loadings. Given the poor initial fit, modification indices were examined, and correlations between several item residuals were added based on both statistical guidance and theoretical considerations. Specifically, error covariances were specified between Items 5 and 6, Items 5 and 7, Items 6 and 7, and Items 1 and 7. Following these modifications, model fit improved substantially. The final model showed a nonsignificant Chi‐square test (χ^2^ = 11.8, df = 10, p = 0.299), with excellent values for CFI (0.991) and TLI (0.981), and acceptable SRMR (0.051). The RMSEA was 0.051 (90% CI = 0.00–0.145). Although the RMSEA point estimate indicates acceptable fit, the upper bound of the confidence interval exceeds conventional thresholds, suggesting some uncertainty regarding model fit.

The PDQ‐Exercise score showed a moderate positive correlation with MHYS (ρ = 0.441, *p* <0.001) and with the MDS‐UPDRS Total score (ρ = 0.545, *p* <0.001). Moderate correlations were also observed with the MDS‐UPDRS Motor Daily Living (ρ = 0.409, *p* <0.001) and Motor Examination subscales (ρ = 0.489, *p* <0.001). A moderate correlation was found with the Ox‐PAQ Total score (ρ = 0.514, *p* <0.001), as well as with its subdomains, including Routine Activities (ρ = 0.495, p<0.001), Social Engagement (ρ = 0.420, p<0.001), and Emotional Wellbeing (ρ = 0.425, *p* <0.001). Weaker but statistically significant correlations were observed with the MDS‐UPDRS Non‐Motor Symptoms (ρ = 0.313, *p* = 0.008) and Motor Complications (ρ = 0.294, *p* = 0.013) subscales. The PDQ‐Exercise also demonstrated a moderate correlation with the PDQ‐8 (ρ = 0.494, *p* <0.001). Overall, all predefined hypotheses were confirmed, yielding a 100% hypothesis success rate, thereby supporting the construct validity of the PDQ‐Exercise (Table 2).

**TABLE 2:** Correlations between PDQ‐Exercise and other measures for construct validity.

	PDQ‐Exercise		Strength of rho	Hypothesis testing outcome
	rho	p		
**MHYS**	0.441	<0.001*	Moderate	Confirmed
**MDS‐UPDRS Total**	0.545	<0.001*	Moderate	Confirmed
MDS‐UPDRS Non‐Motor Symptoms	0.313	0.008*	Weak	Confirmed
MDS‐UPDRS Motor Daily Living	0.409	<0.001*	Moderate	Confirmed
MDS‐UPDRS Motor Examination	0.489	<0.001*	Moderate	Confirmed
MDS‐UPDRS Motor Complications	0.294	0.013*	Weak	Confirmed
**Ox‐PAQ Total**	0.514	<0.001*	Moderate	Confirmed
Ox‐PAQ Routine Activities	0.495	<0.001*	Moderate	Confirmed
Ox‐PAQ Social Engagement	0.420	<0.001*	Moderate	Confirmed
Ox‐PAQ Emotional Wellbeing	0.425	<0.001*	Moderate	Confirmed
**PDQ‐8**	0.494	<0.001*	Moderate	Confirmed

*Significant at *p* < 0.05.

Abbreviations: MDS‐UPDRS = Movement Disorder Society Unified Parkinson's Disease Rating Scale; MHYS = Modified Hoehn and Yahr Stage; Ox‐PAQ = Oxford Participation and Activities Questionnaire; PDQ‐8 = Parkinson's Disease Questionnaire‐8; PDQ‐Exercise = Parkinson's Disease Questionnaire‐Exercise.

Internal consistency was evaluated using both Cronbach's alpha and McDonald's omega. The results indicated acceptable reliability for the scale (α = 0.783; ω = 0.788), with closely aligned estimates supporting the internal consistency of the measure. Cronbach's alpha was 0.783, and item‐deleted alpha values ranged from 0.722 to 0.774. Item‐deleted McDonald's omega values ranged from 0.744 to 0.785, indicating that no item substantially improved or reduced the scale's reliability when removed. These findings suggest that the Turkish version of the PDQ‐Exercise demonstrates acceptable internal consistency (Table 3).

**TABLE 3:** Internal consistency.

Items	Item‐Total Correlation	Cronbach's α if Item Deleted	McDonald's ω if Item Deleted
Item 1. Felt you have struggled to maintain your exercise regime?	0.522	0.757	0.767
Item 2. Had difficulty doing as much exercise as you feel you should?	0.656	0.722	0.744
Item 3. Felt that your exercise regime is not working?	0.421	0.772	0.785
Item 4. Felt that the amount of exercise you do is never enough?	0.476	0.767	0.772
Item 5. Lacked the motivation to undertake exercise?	0.550	0.747	0.760
Item 6. Lacked the motivation to do the things you enjoy?	0.560	0.745	0.758
Item 7. Had problems moving after exercise?	0.412	0.774	0.782

Total Cronbach's alpha = 0.783, Total McDonald's ω = 0.788.

Of the initial 70 patients who completed the PDQ‐Exercise, 57 returned for re‐assessment within 7–14 days. The ICCs demonstrated excellent test–retest reliability, with a value of 0.985 (95% CI = 0.975–0.991). The standard error of measurement (SEM) was calculated as 2.6, and the minimal detectable difference (MDD) at a 95% confidence level was 7.2.

**FIGURE 1:**
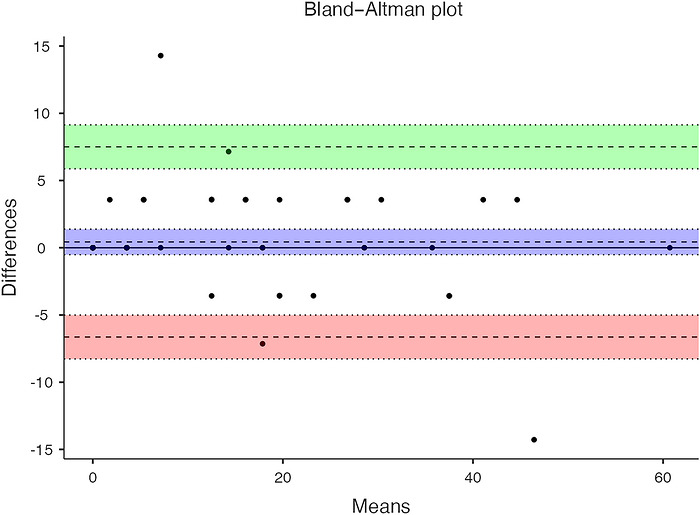
Bland–Altman plots for the PDQ‐Exercise. The means on the *x*‐axis are the average of two trials for the PDQ‐Exercise, and the differences in PDQ‐Exercise scores are on the *y*‐axis.

The Bland–Altman analysis was conducted to assess the agreement between test and retest scores of the PDQ‐Exercise. The mean bias was 0.431 (95%CI: –0.518 to 1.38), indicating minimal systematic difference between the two measurements. The limits of agreement ranged from –6.640 (95%CI: –8.271 to –5.01) to 7.502 (95%CI: 5.872–9.13), suggesting that the majority of score differences fell within an acceptable range (Figure 1). To assess proportional bias, a linear regression analysis was performed with the difference scores as the dependent variable and the mean scores as the independent variable. The results indicated that the mean score did not significantly predict the difference between test and retest measurements (B = –0.041, *p* = 0.203), suggesting no evidence of proportional bias. These findings support the stability and reproducibility of the PDQ‐Exercise over time.

The Turkish version of the PDQ‐Exercise exhibited a significant floor effect. 28.6% of participants scored the minimum score (0) on the total scale. Notably, no participants achieved the maximum possible score on the PDQ‐Exercise.

## 4 Discussion

To date, apart from the original questionnaire development study, no research has addressed the cultural adaptation of the PDQ‐Exercise. This study is the first to investigate the validity and reliability of the Turkish version of the PDQ‐Exercise. The findings demonstrated that the Turkish version of the PDQ‐Exercise demonstrated acceptable psychometric properties in a sample predominantly consisting of individuals with early‐stage PD.

The structural validity of the Turkish version was assessed using CFA. Given that the original study reported a unidimensional structure based on exploratory factor analysis, we also specified a unidimensional model (Morley et al. 2021). However, the initial model fit was inadequate. Modification indices suggested correlating error terms between several pairs of items. The conceptual overlap among the items theoretically justifies these correlations. Items 5 and 6 both tap motivation (general vs. exercise‐specific), and their strong residual correlation likely reflects shared variance not fully captured by the latent factor. Item 7 concerns post‐exercise physical difficulties, but its correlations with Items 5 and 6 suggest that physical challenges and motivation are closely linked, as movement problems may reduce motivation. Likewise, the correlation between Items 1 and 7 likely reflects a common perceived barrier, where physical limitations hinder sustaining exercise.

Although allowing these error covariances improved model fit, such post hoc modifications may indicate local item dependence, redundancy, or semantic overlap rather than true latent structure. Therefore, despite their theoretical plausibility, these correlations should be interpreted cautiously and confirmed in independent samples. In addition, the final model showed acceptable fit based on several indices; however, the RMSEA confidence interval extended beyond conventional thresholds, with the upper bound exceeding 0.10, suggesting some degree of uncertainty in the model fit. Moreover, while the sample size met COSMIN minimum requirements for structural validity, larger samples are generally preferable in CFA to enhance model stability and generalizability (Mokkink et al. 2019). Therefore, the model should be interpreted with caution. Future research should aim to replicate these findings in larger and more diverse samples to improve the stability and generalizability of the factor structure.

Although the correlations observed in the Turkish version were generally lower than those reported in the original validation study (Morley et al. 2021), several factors may account for this discrepancy. First, the characteristics of the present sample likely played an important role. A floor effect was observed in the Turkish version, indicating that a substantial proportion reported minimal exercise‐related difficulties. This, combined with the relatively low disability levels in our sample, suggests limited variability in perceived exercise barriers, a condition that mathematically attenuates correlation coefficients through restriction of range, regardless of the true underlying relationship between constructs. When most participants cluster at the lower end of a scale, the statistical power to detect associations with other measures is inherently reduced. Second, an important methodological difference between the two studies should be noted. The present study required participants to have been engaged in regular exercise for at least 1 month prior to inclusion, whereas no such criterion was applied in the original validation study (Morley et al. 2021). This means our sample was, by design, composed of more physically active individuals who may perceive fewer exercise‐related difficulties than a general Parkinson's disease population. This selection effect likely compressed the range of scores and further contributed to the weaker correlations observed. Finally, cultural differences in the perception, terminology, and reporting of exercise may have played a role. In the Turkish context, physical activity is often less clearly distinguished from general daily movement, and exercise as a deliberate, structured behavior may carry different salience or meaning compared to English‐speaking populations where the original scale was developed. Sociocultural norms around chronic illness management, including a greater tendency toward physician‐directed activity decisions and lower rates of self‐initiated exercise participation, may have attenuated the relationship between perceived exercise difficulty and broader outcomes such as quality of life and participation. Taken together, these factors, particularly the floor effect, low disability levels, and the exercise inclusion criterion, likely account for the relatively lower correlation coefficients observed in the present study. Nevertheless, the overall pattern of associations remains consistent with theoretical expectations, and all predefined hypotheses were confirmed, thereby providing strong support for the construct validity of the Turkish PDQ‐Exercise.

Importantly, the current study extended the construct validation process by incorporating clinical severity measures not included in the original study (Morley et al. 2021). The PDQ‐Exercise showed weak‐to‐moderate correlations with MDS‐UPDRS Total (ρ = 0.545), Motor Examination (ρ = 0.489), and Motor Daily Living subscales (ρ = 0.409), as well as with the MHY Scale (ρ = 0.441). These additional correlations suggest that greater perceived exercise‐related difficulty is associated with more severe motor impairment and disease stage, thereby expanding the construct validity of the PDQ‐Exercise to include clinically relevant indicators of PD severity.

Although the correlations were overall weaker than those reported in the original English version, this may be partly attributed to differences in sample characteristics, or cultural perceptions of exercise (Morley et al. 2021). Nevertheless, the pattern of associations, particularly with motor severity, functional status, and participation, supports the construct validity of the Turkish PDQ‐Exercise. Future research should include more diverse samples with higher symptom burden and greater variability in exercise behavior to further explore the scale's sensitivity and clinical utility.

The internal consistency of the Turkish version of the PDQ‐Exercise was acceptable, with a Cronbach's alpha of 0.783 and McDonald's omega of 0.788, which is lower than the Cronbach's alpha value (α = 0.89) reported in the original study (Morley et al. 2021). Item deletion analysis indicated that removing any individual item reduced the overall alpha, and therefore, no item was considered for removal. Although the internal consistency does not reach the level reported in the original version, it remains within acceptable limits, supporting the reliability of the scale as a unidimensional measure.

Test–retest reliability, not reported in the original study (Morley et al. 2021), was assessed over a 7–14‐day interval in 57 participants. The ICC for the total PDQ‐Exercise score was 0.985, indicating excellent stability. The SEM was 2.6 and the MDD95 was 7.2, suggesting that changes greater than 7.2 points reflect true change beyond measurement error. Bland–Altman analysis showed randomly distributed differences with no proportional bias, supporting good agreement. The nonsignificant regression analysis supports the absence of proportional bias, suggesting that differences between test and retest scores were not dependent on the magnitude of the measurements. Together, these findings demonstrate excellent reproducibility of the Turkish PDQ‐Exercise and provide the first evidence of its test–retest reliability, strengthening the scale's overall psychometric profile.

A floor effect, but no ceiling effect, was observed in the Turkish version, with 28.6% of participants scoring at the minimum on the total score. This indicates that a substantial proportion of participants reported minimal exercise‐related difficulties, likely reflecting the relatively good physical condition and low perceived barriers. Although the original study did not report floor or ceiling effects for the total score, six of the seven items exhibited floor effects (>15%), suggesting a similar pattern of response distribution (Morley et al. 2021).

The magnitude of the observed floor effect represents a notable limitation, as it may indicate restricted score variability and a skewed distribution. This can reduce the sensitivity of the scale to detect differences between individuals, particularly among those with mild symptoms, and may also limit its responsiveness to change over time. Consequently, the instrument may be less suitable for monitoring subtle changes or for use in populations with low levels of exercise‐related difficulties. Despite this limitation, the Turkish version demonstrated acceptable validity and reliability within the studied sample. Future studies should include more heterogeneous samples, particularly individuals with moderate‐to‐severe disease, to further evaluate the scale's measurement properties, responsiveness, and distributional characteristics across the full spectrum of disease severity.

Beyond the original English version, the PDQ‐Exercise has been translated into Brazilian Portuguese, German, and Australian English, although psychometric evaluations are still ongoing. To our knowledge, this is the first study to examine the PDQ‐Exercise in a language other than the original. Demonstrating the validity and reliability of the Turkish version supports its cross‐cultural applicability and broader international use in global health research.

The Turkish PDQ‐Exercise has meaningful potential for clinical application in physiotherapy and rehabilitation practice. As the first PROM specifically designed to capture exercise‐related challenges in people with PD, it enables clinicians to move beyond motor assessments and to understand patients' subjective difficulties with maintaining exercise regimes, motivational deficits, and physical problems following exercise, information that is rarely captured by standard clinical tools. In rehabilitation settings, the scale could be used at baseline to tailor individualized exercise programs, and at follow‐up to monitor changes in exercise‐related challenges over time, thereby supporting patient‐centered goal setting and shared decision‐making. Its brevity and self‐administered format make it particularly practical for routine use in outpatient neurological physiotherapy without adding significant burden to clinical consultations. Given that the sample in this study predominantly consisted of individuals with early‐stage PD, the PDQ‐Exercise may be especially valuable in early rehabilitation intervention, a period where promoting and sustaining exercise engagement is critical for long‐term disease management. Future research should explore its responsiveness to exercise‐based interventions and its utility in tracking rehabilitation outcomes across different stages of PD.

This study has some limitations. First, participants were recruited from a single outpatient clinic, which may introduce recruitment bias and limit the generalizability of the findings to broader clinical and community‐based populations. Second, most participants were in the early stages of PD, indicating relatively good physical condition and fewer exercise‐related challenges. As a result, many participants selected the lowest response option on the scale, reflecting minimal perceived barriers to exercise. This may have contributed to the observed floor effects. Such effects can reduce score variability and limit the scale's ability to detect differences or changes, particularly among individuals with more advanced disease. Consequently, the generalizability of these findings to patients with more severe motor and physical impairments is limited. Future studies should include more diverse and representative samples, including individuals across different disease stages and from multiple centers, to better capture the full spectrum of exercise‐related difficulties. An additional limitation relates to the inclusion criterion requiring at least 1 month of prior exercise therapy. Although this criterion was applied to ensure that participants had sufficient experience to meaningfully evaluate exercise‐related barriers, it may have introduced selection bias toward more active and motivated individuals. Consequently, participants may have perceived fewer barriers to exercise, which could partly explain the observed floor effects. Therefore, the findings may not fully reflect the experiences of more sedentary people with PD or those without prior exposure to structured exercise programs. Future studies should consider including participants with varying levels of exercise experience to enhance representativeness. A limitation of this study is that content validity was not quantified using a content validity index (CVI). Although content validity was evaluated qualitatively through expert committee review, and full agreement was reached, the absence of a quantitative CVI limits the ability to formally document the level of expert agreement.

## 5 Conclusion

The findings suggest that the Turkish version of the PDQ‐Exercise demonstrated generally acceptable psychometric properties in a sample predominantly consisting of individuals with early‐stage PD; however, further studies are needed to evaluate its responsiveness, known‐groups validity, and measurement invariance across diverse populations. As the first PROM specifically designed to evaluate exercise‐related challenges in this population, the PDQ‐Exercise represents an important innovation in both research and rehabilitation practice. Its brief, self‐administered format offers practical advantages for clinical settings, making it particularly suitable for routine use in outpatient neurological physiotherapy without adding significant burden to clinical consultations. Clinicians can use it at baseline to tailor individualized exercise programs and at follow‐up to monitor changes in exercise‐related challenges over time, supporting patient‐centered goal setting and shared decision‐making in rehabilitation.

## Author Contributions

**Melis Yalçın**: conceptualization, investigation, writing – original draft, methodology, writing – review and editing, data curation. Turhan Kahraman: conceptualization, investigation, writing – original draft, methodology, writing – review and editing, formal analysis, supervision. **Arzu Genç**: conceptualization, investigation, writing – review and editing, supervision, methodology. **Berril Dönmez Çolakoğlu**: conceptualization, investigation, methodology, resources, data curation, writing – review and editing.

## Funding

The authors have nothing to report.

## Ethical Approval

The study protocol was approved by the Non‐Invasive Research Ethics Committee of Dokuz Eylul University (protocol number: 7071‐GOA).

## Conflicts of Interest

The authors declare no conflicts of interest.

## Informed Consent Statement

All participants provided written informed consent before participating in the study.

## Supporting information



Supporting Information: brb371447‐sup‐0001‐SuppMat.docx

## Data Availability

Date sets are not available publicly because of legal/security/privacy/policy reasons. However, it is available upon request from the corresponding author.
