# Supplementary material for: Cross‐Cultural Adaptation and Psychometric Evaluation of the Turkish Version of the Exercise‐Specific Parkinson's Disease Questionnaire
Source: Brain Behav. 2026 Jun 10;16(6):e71447. doi: 10.1002/brb3.71447 (PMC13250624; doi:10.1002/brb3.71447)
Supplement: Supplementary file 1 — Supporting Information: brb371447‐sup‐0001‐SuppMat.docx [file BRB3-16-e71447-s001.docx]

**Supplementary Material: Cognitive Debriefing Sample Characteristics**

This supplementary document provides detailed demographic and clinical information for participants included in the cognitive debriefing phase of the Turkish adaptation of the PDQ-Exercise.

A total of 15 people with Parkinson’s disease participated in the cognitive debriefing interviews. Participants were selected to ensure variability in age, gender, disease duration, and educational background.

**Demographic and Clinical Characteristics of Participants**

- **Age range:** 52–84 years
- **Gender distribution:** Both male and female participants were included
- **Disease duration:** 3–16 years
- **Educational level:** Ranged from primary school to university graduates
- **Occupational background:** Included retired individuals, homemakers, manual workers, and professionals
- **Marital status:** Included married, single, and widowed participants

**Summary of Individual Participant Characteristics**

1. 75-year-old male, disease duration 4 years, high school graduate, retired, married
2. 66-year-old male, disease duration 12 years, university graduate, retired teacher, married
3. 75-year-old male, disease duration 5 years, primary school graduate, retired chef, married
4. 77-year-old male, disease duration 13 years, university graduate, retired literature teacher, widowed
5. 53-year-old male, disease duration 3 years, primary school graduate, construction worker, married
6. 82-year-old male, disease duration 7 years, university graduate, upholstery worker, married
7. 58-year-old female, disease duration 5 years, primary school graduate, self-employed, single
8. 68-year-old female, disease duration 10 years, primary school graduate, retired factory worker, single
9. 75-year-old female, disease duration 16 years, primary school graduate, retired, widowed
10. 64-year-old female, disease duration 10 years, primary school graduate, housewife, married
11. 84-year-old male, disease duration 11 years, primary school graduate, retired (aviation sector), widowed
12. 68-year-old male, disease duration 6 years, university graduate, retired accountant, married
13. 70-year-old male, disease duration 6.5 years, university graduate, retired banker, married
14. 52-year-old female, disease duration 3 years, primary school graduate, housewife, married
15. 52-year-old female, disease duration 5 years, primary school graduate, market vendor, married

**Purpose of the Sample**

The sample was intentionally diverse to ensure that the translated instrument was evaluated across a wide range of demographic and clinical profiles. This allowed for a comprehensive assessment of item clarity, interpretability, and cultural relevance. The variability in educational level and clinical characteristics was particularly important for identifying potential comprehension issues and ensuring the accessibility of the questionnaire across different subgroups of people with Parkinson’s disease.
